# Retinoic acid receptor alpha inhibits ferroptosis by promoting thioredoxin and protein phosphatase 1F in lung adenocarcinoma

**DOI:** 10.1038/s42003-024-06452-7

**Published:** 2024-06-20

**Authors:** Yunyi Bian, Guangyao Shan, Jiaqi Liang, Zhengyang Hu, Qihai Sui, Haochun Shi, Qun Wang, Guoshu Bi, Cheng Zhan

**Affiliations:** Department of Thoracic Surgery, Zhongshan Hospital, Fudan University, Shanghai, China

**Keywords:** Non-small-cell lung cancer, Molecular biology

## Abstract

Ferroptosis is a recently discovered form of cell death that plays an important role in tumor growth and holds promise as a target for antitumor therapy. However, evidence in the regulation of ferroptosis in lung adenocarcinoma (LUAD) remains elusive. Here, we show that retinoic acid receptor alpha (*RARA*) is upregulated with the treatment of ferroptosis inducers (FINs). Pharmacological activation of *RARA* increases the resistance of LUAD to ferroptosis according to cell viability and lipid peroxidation assays, while *RARA* inhibitor or knockdown (KD) does the opposite. Through transcriptome sequencing in *RARA*-KD cells and chromatin immunoprecipitation (CHIP)-Seq data, we identify thioredoxin (*TXN*) and protein phosphatase 1 F (*PPM1F*) as downstream targets of *RARA*, both of which inhibit ferroptosis. We confirm that RARA binds to the promotor region of *TXN* and *PPM1F* and promotes their transcription by CHIP-qPCR and dual-luciferase assays. Overexpression of *TXN* and *PPM1F* reverses the effects of *RARA* knockdown on ferroptosis in vitro and vivo. Clinically, *RARA* knockdown or inhibitor increases cells’ sensitivity to pemetrexed and cisplatin (CDDP). Immunohistochemistry (IHC) of LUAD from our cohort shows the same expression tendency of RARA and the downstream targets. Our study uncovers that *RARA* inhibits ferroptosis in LUAD by promoting *TXN* and *PPM1F*, and inhibiting *RARA*-*TXN/PPM1F* axis represents a promising strategy for improving the efficacy of FINs or chemotherapy in the treatment of LUAD patients.

## Abstract (web summary)

This study reveals RARA’s role in inhibiting ferroptosis through the promotion of TXN and PPM1F, offering a potential therapeutic strategy for LUAD treatment by increasing efficacy of ferroptosis inducers or chemotherapy.

## Introduction

Lung cancer represents the most prevalent malignancy and the primary cause of cancer-related mortality globally^1^. Non-small cell lung cancer (NSCLC) constitutes 82% of lung cancer cases^2^, with lung adenocarcinoma (LUAD) being the predominant subtype^3^. Despite current treatment modalities, the five-year survival rate of LUAD remains suboptimal^4,5^. Thus, exploring the molecular mechanisms underlying LUAD progression and identifying novel therapeutic targets are urgently needed.

Ferroptosis is a distinct form of regulated cell death that differs from apoptosis and other types of cell death^6,7^. It is characterized by an imbalance of reactive oxygen species (ROS) production^8^, which catalyzes the peroxidation of polyunsaturated fatty acids (PUFA) on the biological membrane in the presence of intracellular iron overload via the Fenton reaction^9,10^. In cancer cells, ROS always accumulated at a high level and was a critical factor in ferroptosis sensitivity^11^, as well as intracellular iron level^12^. The glutathione peroxidase 4 (GPX4) system, which relies on glutathione (GSH), is the most crucial intracellular mechanism against ferroptosis^13^. Commonly used ferroptosis inducers (FINs) such as erastin and RSL3 trigger ferroptosis by targeting the GPX4-dependent GSH system^14,15^. In recent years, studies based on the application prospect of ferroptosis inducers (FINs)^16^ in tumor treatment have gradually deepened. FIN could increase the efficacy of conventional therapies such as radiotherapy^17^, chemotherapy^18^, and targeted therapy^19^. When combined with the chemotherapy drug cisplatin (CDDP), erastin is more effective in treating NSCLC than cisplatin alone[18, 20].

Retinoic acid receptor alpha (RARA) is a member of the RAR family and is a transcription factor (TF) regulated by retinoic acid (RA)^20^. The *RARA* signaling pathway plays a crucial role in growth and remodeling in tissue homeostasis and participates in the regulation of multiple crucial biological pathways, such as growth, differentiation, reproduction, and tumor progression^21^. Previous studies have shown that the *RARA* pathway is associated with the proliferation and metastasis of lung cancer^22^ and with resistance to all-trans-retinoic acid (ATRA) therapy^23,24^. Furthermore, *RARA* signaling is critical in antioxidant function^25,26^ and is involved in regulating iron metabolism imbalance through transcriptional regulation^27,28^. Because of the significance of iron and oxidative stress in ferroptosis initiation, it is important to investigate the unclear connection between RARA and ferroptosis to enhance the role of this vital TF in cancer therapy.

Through transcriptome sequencing, we found that *RARA* expression increased significantly in LUAD cells after FIN treatment, suggesting that RARA may act as an essential mediator in ferroptosis. In this study, we discovered that *RARA* inhibited LUAD ferroptosis both in vitro and in vivo. With the combination of RNA-Seq and ChIP-seq, we systematically revealed that RARA was a transcription promotor for thioredoxin (*TXN*) and protein phosphatase 1 F (*PPM1F*), both of which were critical suppressors of ferroptosis^29–31^. TXN is a protein that participates in numerous redox reactions within cells. Previous research has demonstrated its involvement in regulating inflammation and inhibiting apoptosis^32^. It has also been reported to inhibit ferroptosis by modulating the activity of GPX4 and GSH levels^29^. Protein PPM1F is a member of the PP2C family of Ser/Thr protein phosphatases and was demonstrated to dephosphorylate and negatively regulate PAK^30,33,34^, which promotes the occurrence of ferroptosis by enhancing Yes-associated protein (YAP) activity^31^. Furthermore, we demonstrated that the knockdown of *RARA* stimulated the ferroptosis of LUAD and enhanced the efficacy of chemotherapy pemetrexed and CDDP. Our findings suggest that targeting the *RARA-TXN/PPM1F* pathway may represent a novel therapeutic strategy for treating LUAD.

## Results

### Transcriptome sequence suggests *RARA* as a cardinal ferroptosis mediator

**Fig. 1:**
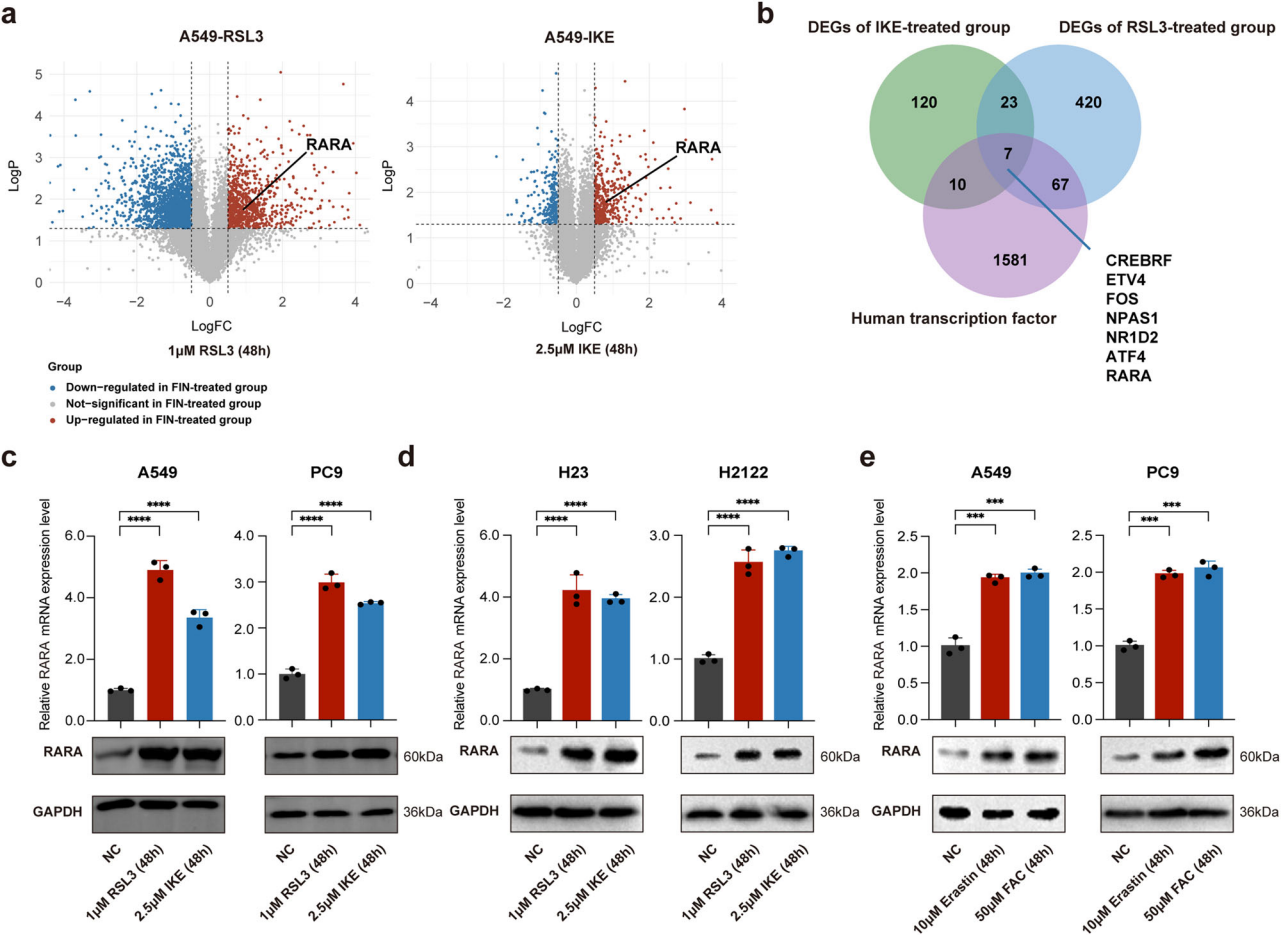
Identification of RARA related to ferroptosis. **a** The volcano plot showed the differentially expressed genes in the FIN-treated group vs. the non-treated group, and RARA was upregulated in the FIN-treated group (A549 cell: RSL3 1 μM and IKE 2.5 μM for 48 h). **b** Venn plot displayed the ferroptosis-related transcription factors by the intersection of upregulated DEGs in RSL3-treated and IKE-treated groups in A549 cells. **c**, **d** qRT-PCR and WB verified the expression of RARA in the FIN-treated group vs. the non-treated group in A549, PC9, H23, and H2122 cells. (*n* = 3 biologically independent experiments, Student *t*-test). **e** qRT-PCR and WB showed the upregulation of RARA by other FINs (erastin 10 μM and FAC 50 μM for 48 h) (*n* = 3 biologically independent experiments, Student *t*-test) *p < 0.05; **p < 0.01; ***p < 0.001; ****p < 0.0001.

Firstly, we treated A549 cells with the ferroptosis inducers RSL3 (1 μM) and IKE (2.5 μM) for 48 h, and observed a significant upregulation of the seven transcription factors in both FIN-treated groups compared to non-treated cells by RNA sequencing analysis (Fig. 1a, b). Considering the antioxidant and iron-modulating function, we validated the upregulated expression of *RARA* in mRNA and protein levels in four lung cancer cell lines (Fig. 1c, d). Also, the upregulation of *RARA* was induced by two other FINs, including erastin (10 μM, 48 h) and FAC (50 μM, 48 h) (Fig. 1e). According to the above results, ferroptosis activates the expression of *RARA*, suggesting the unexpected relationship between *RARA* and ferroptosis.

### Pharmacological or genetic alteration of *RARA* changes sensitivity to ferroptosis in LUAD cells

**Fig. 2:**
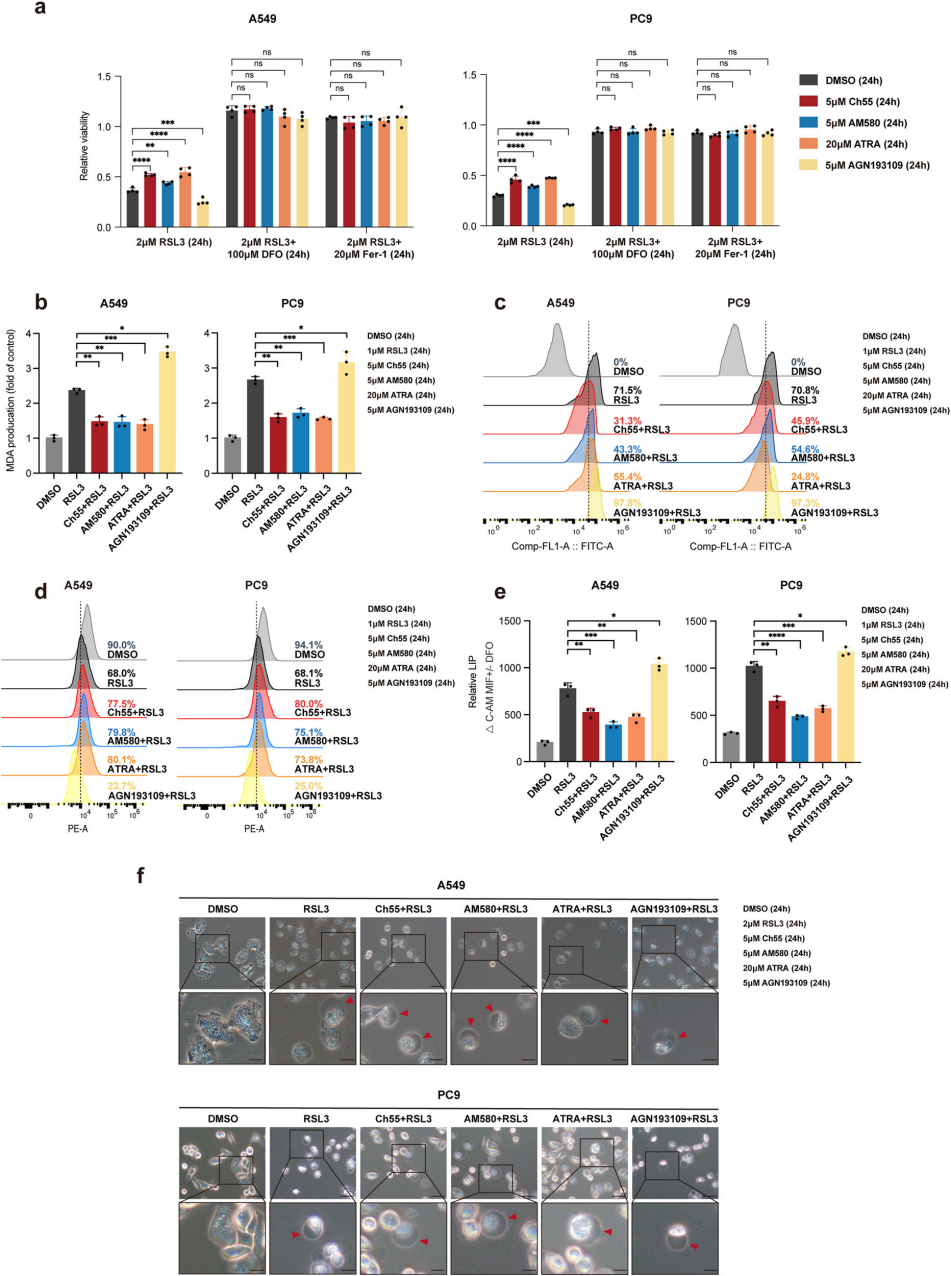
Pharmacological activation or inhibition of RARA changes sensitivity to ferroptosis in LUAD cells. **a** CCK8 assays to detect the cell viability of A549 and PC9 cells treated with RSL3 (2 μM), RSL3 (2 μM) plus DFO (100 μM) or fer-1 (20 μM) for 24 h after incubating with DMSO, Ch55 (5 μM), AM580 (5 μM) ATRA (20 μM) or AGN193109 (5 μM) for 24 h. (*n* = 4 biologically independent experiments, Student *t*-test). **b**–**e** MDA level (**b**) lipid peroxidation (**c**), mitochondrial membrane potential (MMP) by TMRE (**d**), and labile iron pool (LIP) by CA-AM (**e**) were accessed in A549 and PC9 cells treated with DMSO or RSL3 (1 μM) for 24 h after incubating with DMSO, Ch55 (5 μM), AM580 (5 μM) ATRA (20 μM) or AGN193109 (5 μM) for 24 h. (*n* = 3 biologically independent experiments, Student *t*-test). **f** Light microscopy images showed the degrees of “ballooning phenotype” in A549 and PC9 cells treated with DMSO or RSL3 (1 μM) for 24 h after incubating with DMSO, Ch55 (5 μM), AM580 (5 μM) ATRA (20 μM) or AGN193109 (5 μM) for 24 h. Scale bar:50 μm. The zoomed-in figures, scale bar: 250 μm. ns, not significant; *p < 0.05; **p < 0.01; ***p < 0.001; ****p < 0.0001.

To further understand the role of the *RARA* pathway in ferroptosis, we pretreated both cell lines with *RARA* activators and the inhibitor for 24 h and investigated the alteration of cells’ sensitivity to FINs (24 h). Our results demonstrated that specific agonists Ch55 (5 μM) and AM580 (5 μM), as well as the direct agonist ATRA (20 μM), conferred resistance to RSL3 (2 μM) in both A549 (*p* < 0.01) and PC9 (*p* < 0.001) cells, with ATRA having the most pronounced effect. Conversely, pre-treatment with the pan-RAR antagonist AGN193109 (5 μM) sensitized cells to RSL3 (2 μM). This effect could be reversed by the ferroptosis inhibitors DFO (100 μM) or ferrostatin-1 (20 μM) (Fig. 2a) but not by the apoptosis inhibitor Z-VAD-FMK (10 μM) or the necroptosis inhibitor necrosulfonamide (1 μM) (Supplementary Fig. 1b). Similar results were obtained upon treatment with IKE (5 μM) (Supplementary Fig. 1a-b). Next, we assessed ferroptosis degrees in different treatment groups. Pre-treatment with agonists decreased the MDA (Fig. 2b) and lipid peroxidation (Fig. 2c) levels in both A549 and PC9 cells, while AGN193109 generated the opposite effect. As for the measurement of mitochondrial membrane potential (MMP, Δψ), *RARA* activators increased Δψ, indicating mitochondrial hyperpolarization, and AGN193109 decreased it, which showed mitochondrial dysfunction (Fig. 2d). Furthermore, the labile iron pool (LIP) level was measured by the calcein-acetoxymethyl ester (CA-AM) method. Activation of *RARA* decreased the LIP level, and *RARA* inhibition enhanced the LIP level (Fig. 2e). The ballooning phenotype was decreased with *RARA* activators pre-treatment and increased with AGN193109 for 24 h (Fig. 2f).

**Fig. 3:**
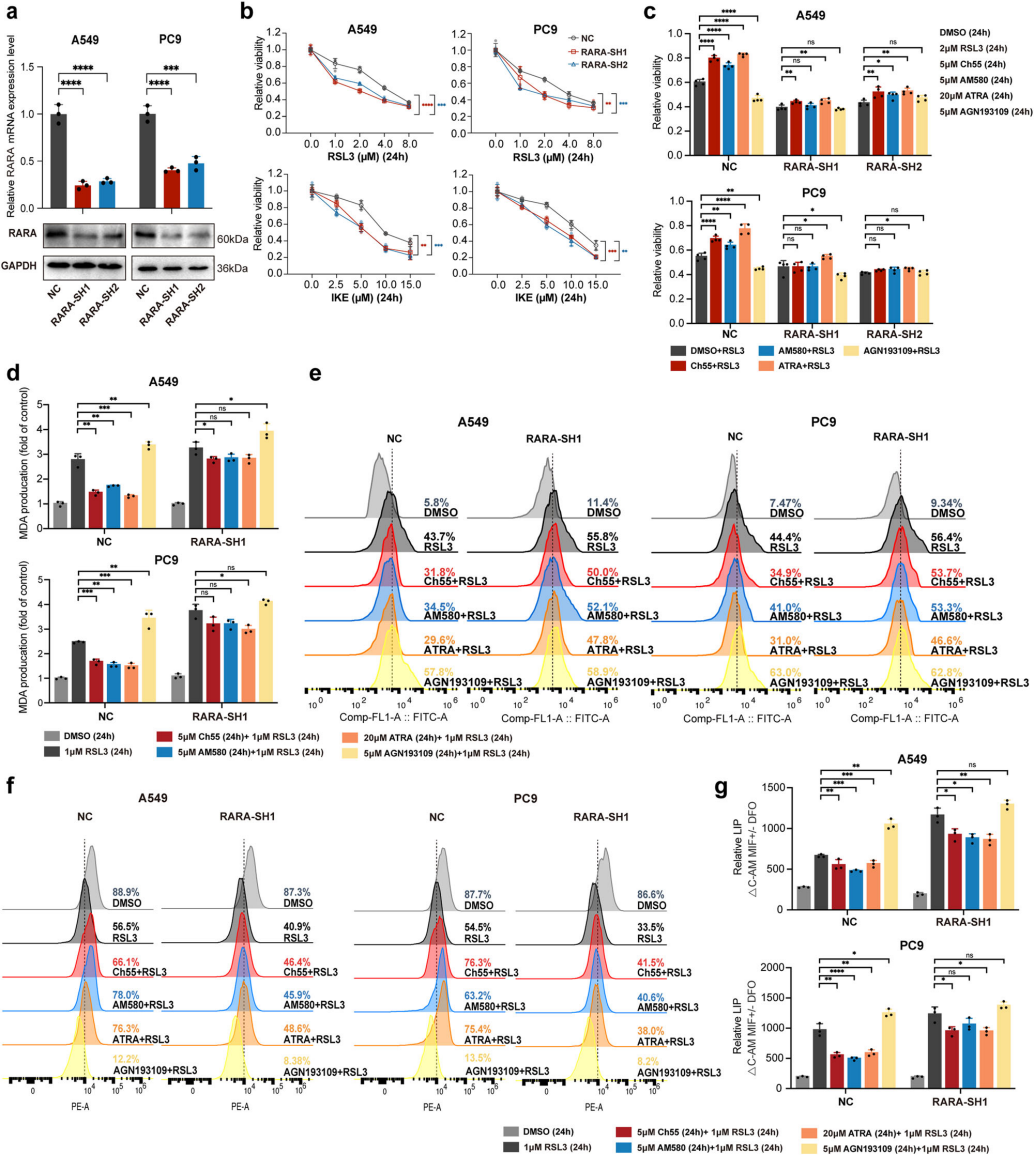
Genetic silence of RARA changes sensitivity to ferroptosis in LUAD cells. **a** qRT-PCR and WB assays confirmed the mRNA and protein expression level of RARA in A549 and PC9 cells after transfection with NC (control shRNA), RARA-SH1, or RARA-SH2 lentivirus. (*n* = 3 biologically independent experiments, Student *t*-test) (**b**) CCK8 assays to detect the cell viability of NC, RARA-SH1, or RARA-SH2 groups in A549 and PC9 cells treated with a gradient dose of RSL3 or IKE for 24 h. (*n* = 4 biologically independent experiments, two-way ANOVA) (**c**) CCK8 assays to detect the cell viability of NC, RARA-SH1, or RARA-SH2 groups in A549 and PC9 cells treated with DMSO or RSL3 (2 μM) for 24 h after incubating with DMSO, Ch55 (5 μM), AM580 (5 μM) ATRA (20 μM) or AGN193109 (5 μM) for 24 h. (*n* = 4 biologically independent experiments, Student *t*-test). **d**–**g** MDA level (**d**), lipid peroxidation (**e**), MMP by TMRE (**g**) and LIP by CA-AM (**g**) were accessed in NC, RARA-SH1 groups of A549 and PC9 cells treated with DMSO or RSL3 (1 μM) for 24 h after incubating with DMSO, Ch55 (5 μM), AM580 (5 μM) ATRA (20 μM) or AGN193109 (5 μM) for 24 h. (*n* = 3 biologically independent experiments, Student *t*-test) ns, not significant *p < 0.05; **p < 0.01; ***p < 0.001;****p < 0.0001.

We further identified the role of *RARA* in this process by knocking *RARA* in LUAD cell lines using siRNAs (Supplementary Fig. 1c). Cytotoxicity assays demonstrated that *RARA* knockdown (KD) sensitized A549 and PC9 cells to RSL3 and IKE (Supplementary Fig. 1d). Next, we designed lentiviruses containing shRNA sequences targeting *RARA* and transduced them into two cell lines to generate stable *RARA*-KD clones (Fig. 3a). Similarly, *RARA*-KD A549 and PC9 cells became more sensitive to ferroptosis induction by RSL3 and IKE (Fig. 3b). The ferroptosis-desensitizing effects, including MDA and lipid peroxidation decrease caused by Ch55, AM580, and ATRA, were abrogated by *RARA* KD, thus excluding the unspecific *RARA*-independent effect of these compounds in the regulation of ferroptosis, and the *RARA*-KD induced ferroptosis more effectively (Fig. 3c–e). Similar results were also verified in the MMP and LIP assays. The Δψ level decreased, and the LIP level enhanced by *RARA*-KD, indicated more ferroptosis with *RARA* genetic inhibition. Also, the increase of MMP and the decrease of LIP by *RARA* activators were impaired by *RARA*-KD (Fig. 3f, g). The ballooning phenotype verified the effects (Supplementary Fig. 1e).

### Knockdown of *RARA* downregulates the expression of ferroptosis-related genes *TXN* and *PPM1F*

**Fig. 4:**
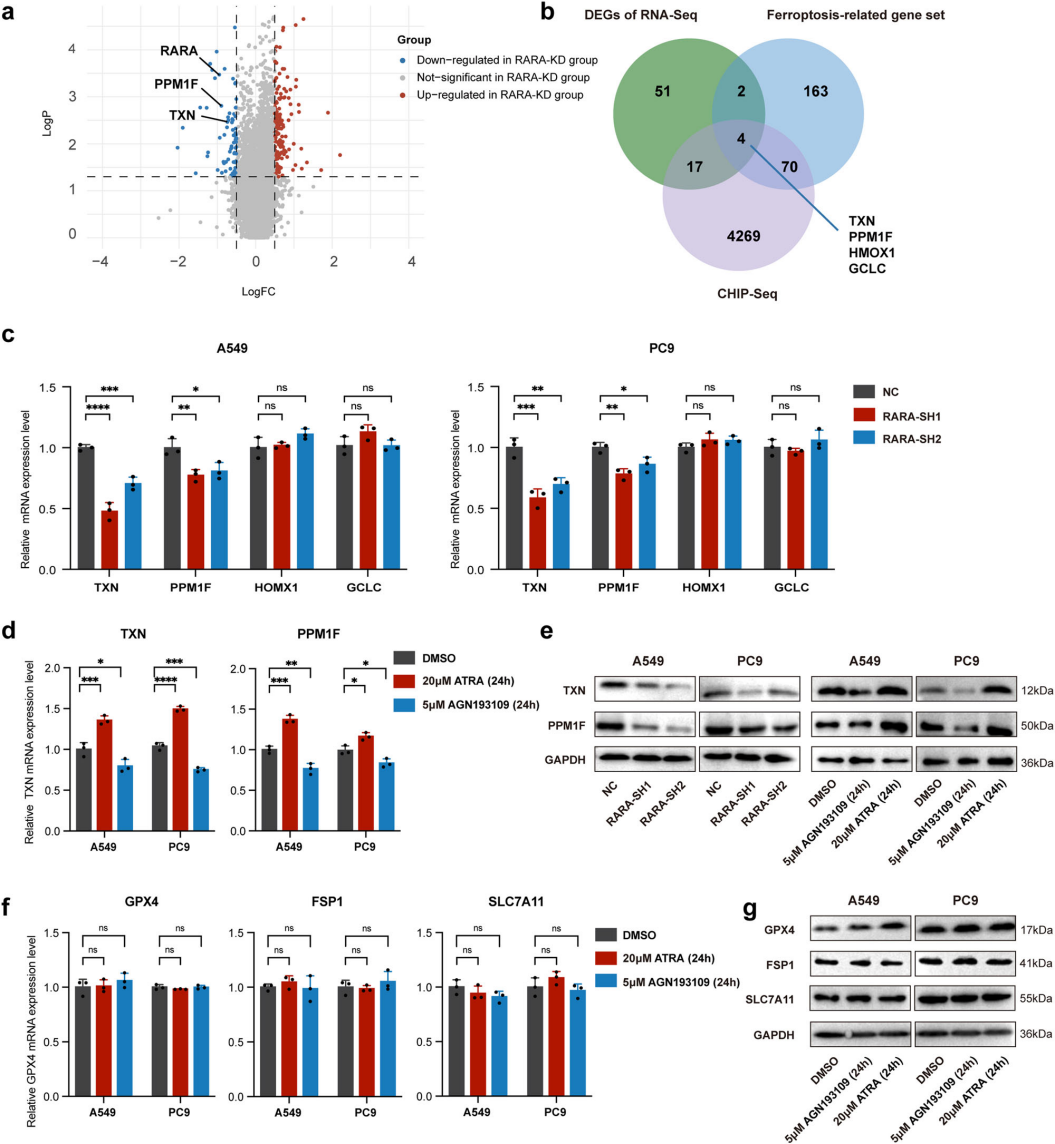
Identification of TXN and PPM1F as downstream targets of RARA. **a** The volcano plot showed the differentially expressed genes in the NC group vs. the RARA-KD group and showed that TXN and PPM1F were down-regulated in the RARA-knockdown group. **b** Venn plot displayed the predicted targets of RARA by the intersection of DEGs of RNA-Seq, CHIP-Seq, and ferroptosis-related gene set. **c** qRT-PCR verified the expression of key downstream genes: TXN, PPM1F, HMOX1, and GCLC in the NC group vs. the RARA-KD group in two cell lines. (*n* = 3 biologically independent experiments, Student *t*-test). **d** qRT-PCR revealed the mRNA expression of TXN and PPM1F in A549 and PC9 cells after treatment with ATRA (20 μM) or AGN193109 (5 μM) for 24 h. (*n* = 3 biologically independent experiments, Student *t*-test). **e** WB showed the protein level of TXN and PPM1F in the NC group vs. the RARA-knockdown group in A549 and PC9 cells as well as the two cells after treatment with ATRA (20 μM) or AGN193109 (5 μM) for 24 h. **f** qRT-PCR showed the expression level of GPX4, FSP1, and SLC7A11 in A549 and PC9 cells. (*n* = 3 biologically independent experiments, Student *t*-test). **g** WB showed the protein level of GPX4, FSP1, and SLC7A11 in A549 and PC9 cells treated with ATRA (20 μM) or AGN193109 (5 μM) for 24 h. ns, not significant; *p < 0.05; **p < 0.01; ***p < 0.001; ****p < 0.0001.

As a transcription factor, RARA primarily promotes gene transcription. Thus, to further elucidate the mechanism by which *RARA* inhibits ferroptosis, we performed RNA-seq analysis to identify the downregulated genes in *RARA*-KD A549 cells. (Fig. 4a). By intersecting these genes with ferroptosis-associated genes from the FerrDb database (http://www.zhounan.org/)^35^ and putative RARA targets from the GTRD ChIP-seq dataset (http://www.gtrd.bioumi.org), we identified 4 potential candidate genes, *TXN*, *PPM1F*, *HMOX1*, and *GCLC* (Fig. 4b). In *RARA*-KD A549 and PC9 cell lines, the results of qRT-PCR showed that the *TXN* and *PPM1F* expression was decreased, while that of *HMOX1* and *GCLC* showed no difference (Fig. 4c). Following treatment of the two cell lines with ATRA (20 μM) and AGN193109 (5 μM) for 24 h, we performed qRT-PCR and observed significant upregulation of *TXN* and *PPM1F* in the ATRA group (Fig. 4d), consistent with ATRA-induced changes in gene expression reported in the GEO database^36^. WB exhibited consistent results with qPCR (Fig. 4e). Furthermore, we validated the expression level of crucial ferroptosis factors including *GPX4*, ferroptosis suppressor protein 1 (*FSP1*) and Solute Carrier Family 7 Member 11 (*SLC7A11*), the qPCR (Fig. 4f) and WB (Fig. 4g) results showed no difference after treatment with ATRA (20 μM) or AGN193109 (5 μM) for 24 h. In summary, our results suggest that the transcription factor *RARA* upregulates the expression of *TXN* and *PPM1F* in LUAD.

### RARA promotes *TXN* and *PPM1F* transcription by binding to promoter regions

**Fig. 5:**
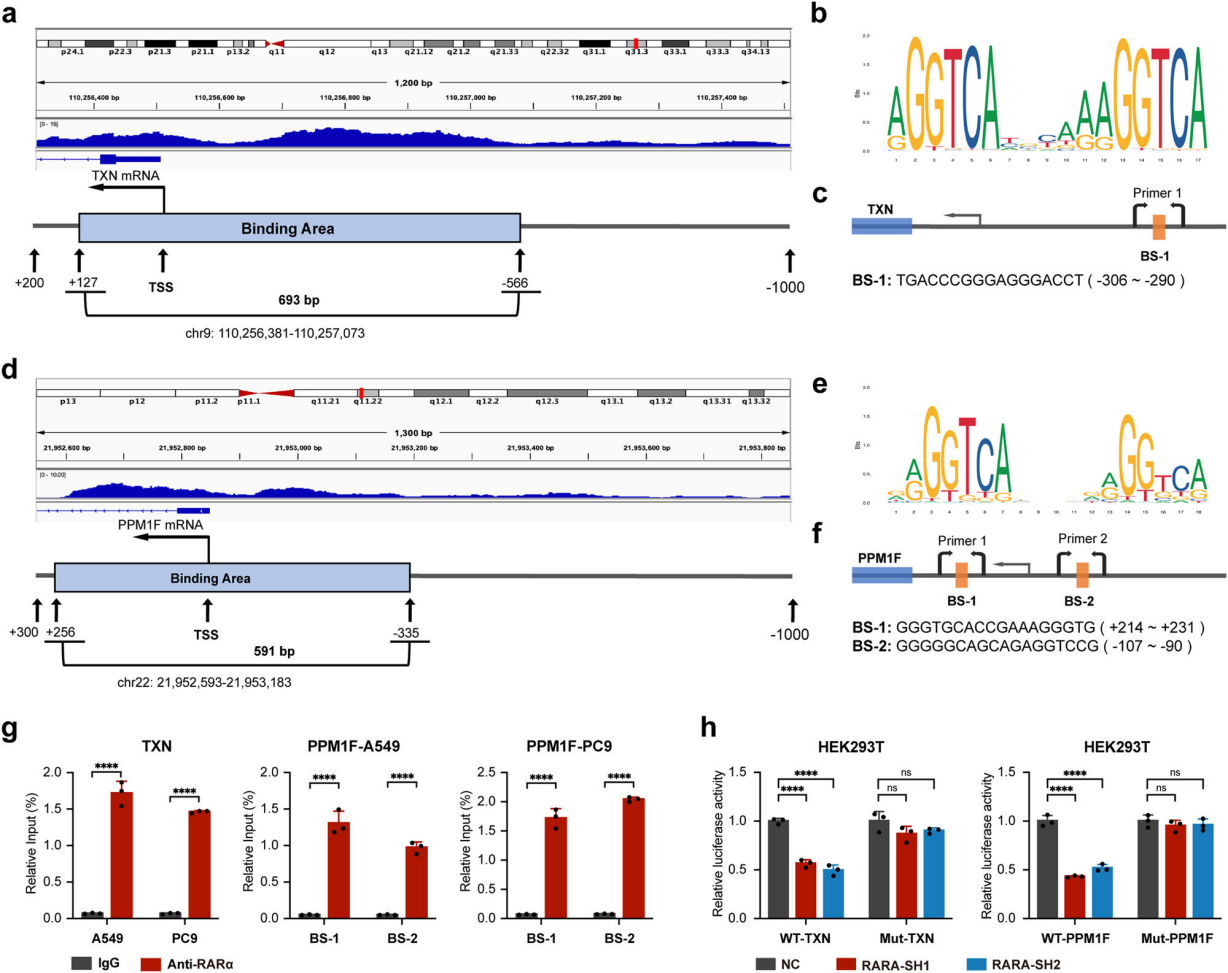
RARA promotes the transcription of TXN and PPM1F by binding to their promotors. **a** Genome-wide data of TXN from the ENCODE database and our CHIP results showed the RARA-binding peak in the promotor region close to the TSS of TXN. **b** Predicted binding sequences of RARA in TXN showed by JASPAR. **c** Predicted BS of RARA in TXN and design of primers for ChIP-qPCR. **d** Genome-wide data of PPM1F from the ENCODE database and our CHIP results showed the RARA-binding peak in the promotor region close to the TSS of PPM1F. **e** Predicted binding sequences of RARA in PPM1F showed by JASPAR. **f** Predicted BS of RARA in PPM1F and design of primers for ChIP-qPCR. **g** ChIP-seq results demonstrated the enrichment of RARA to TXN and PPM1F in A549 and PC9 cells. (*n* = 3 biologically independent experiments, Student *t*-test). **h** Dual-luciferase assays showed the fluorescence intensity of RARA-NC and RARA-KD using luciferase plasmids containing WT or mutated type of the promoter region of TXN and PPM1F. (*n* = 3 biologically independent experiments, Student *t*-test) BS binding sites, ChIP chromatin immunoprecipitation, TSS transcription start site, WT wild type. ns, not significant; *p < 0.05; **p < 0.01; ***p < 0.001; ****p < 0.0001.

Considering that RARA acts as a transcription factor, we consulted the ENCODE database (https://www.encodeproject.org/) and verified the enrichment of RARA in the *TXN* (Fig. 5a) and *PPM1F* (Fig. 5d) promoter region close to the transcription start site (TSS). Next, we selected the putative transcription factor binding sites (TFBS) for *TXN* (Fig. 5b) and *PPM1F* (Fig. 5e) using the JASPAR network resource (https://jaspar.genereg.net/) and then designed corresponding primers for them(Fig. 5c, f). The results of the ChIP experiment showed that RARA can bind to the BS-1 region of *TXN* and BS-1 and BS-2 regions of *PPM1F* promoters (Fig. 5g). These results validate that *TXN* and *PPM1F* are the transcriptional targets of RARA.

Next, a dual-luciferase reporter assay was conducted to confirm that RARA directly regulates the transcription of *TXN* and *PPM1F*. A firefly luciferase reporter plasmid containing the wild-type *RARA* promoter region was constructed, along with a corresponding mutant plasmid in which all putative *RARA* binding sites were randomly altered. In HEK-293T cells transfected with the wild-type plasmid, *RARA* knockdown significantly reduced firefly luciferase activity. This effect could be abrogated by introducing mutations in the *TXN* and *PPM1F* promoter regions (Fig. 5h). The above results reveal the mechanism that RARA promotes the transcription of *TXN* and *PPM1F* by binding to their promotors.

### *TXN* and *PPM1F* reverse the ferroptosis sensitizing effect rendered by *RARA* knockdown

**Fig. 6:**
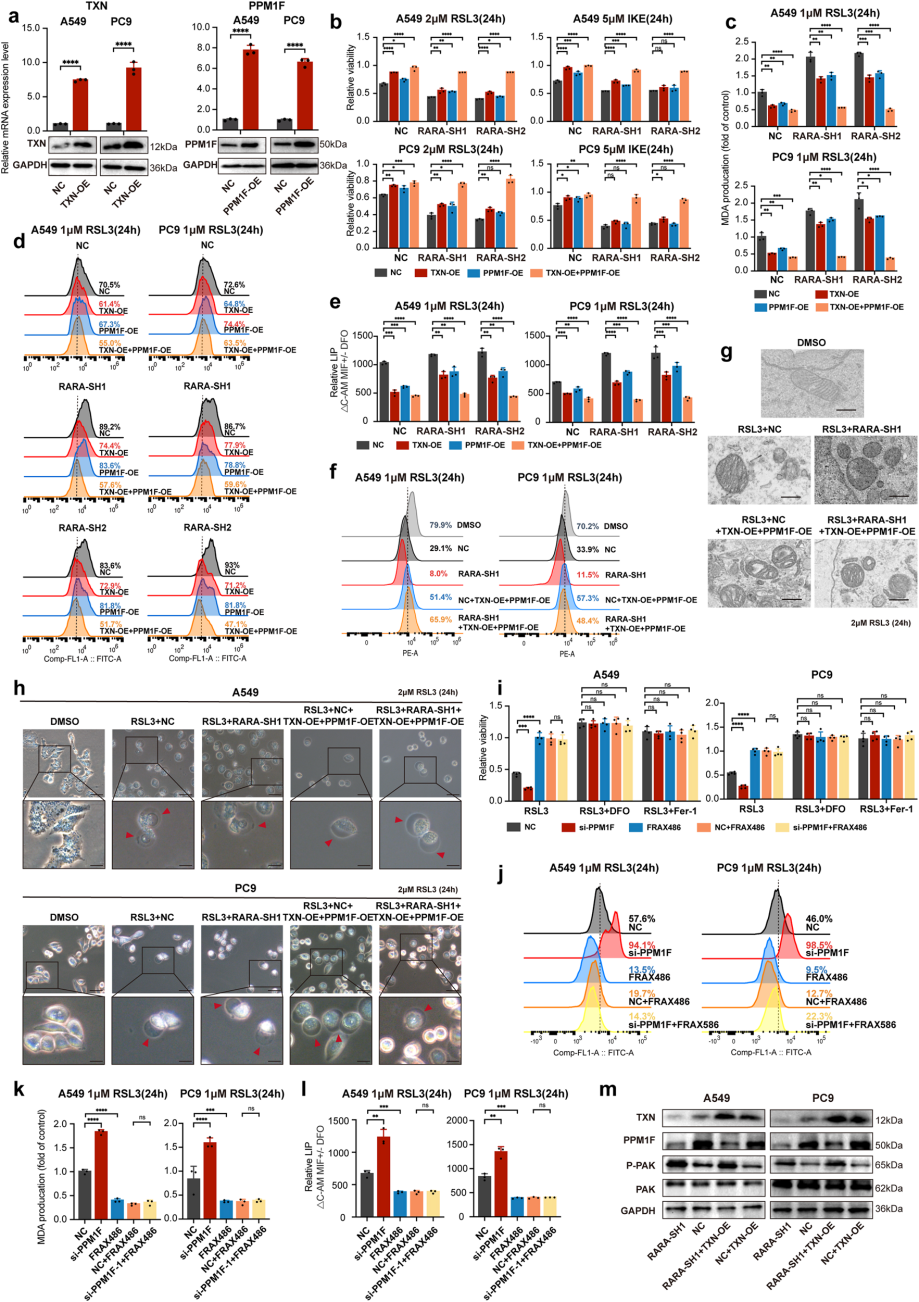
TXN and PPM1F reverses the effect of RARA knockdown on ferroptosis. **a** qRT-qPCR and WB showed the TXN and PPM1F expression when transfecting with vector or TXN-overexpression lentivirus or PPM1F-overexpression lentivirus. (*n* = 3 biologically independent experiments, Student *t*-test). **b** Cell toxicity assays demonstrated the rescue effect of TXN and PPM1F on ferroptosis reduced by RSL3 and IKE (24 h) in RARA-KD A549 and PC9 cells. (*n* = 3 biologically independent experiments, Student *t*-test). **c**–**f** MDA level (**c**), lipid peroxidation level (**d**), LIP level (**e**), and MMP level (**f**) showed the rescue effect of TXN and PPM1F on ferroptosis induced by RSL3 (1 μM, 24 h) in RARA-KD A549 and PC9 cells. (*n* = 3 biologically independent experiments, Student *t*-test). **g** Significant alterations of mitochondria in NC and RARA-knockdown A549 cells with or without TXN-OE and PPM1F-OE. Scale bar:50 nm. **h** Light microscopy images showed “ballooning phenotype” degrees in A549 and PC9 cells with or without TXN-OE and PPM1F-OE treated with DMSO or RSL3 (1 μM, 24 h). Scale bar:50 μm. The zoomed-in figures, scale bar: 250 μm. **i** CCK8 assays showed PPM1F-KD A549 and PC9 cells were more sensitive to RSL3 (2 μM, 24 h), while the PAK phosphorylation inhibitor FRAX486 (0.5 μM, 24 h) had the opposite effect, which can reverse the sensitivity. (*n* = 4 biologically independent experiments, Student *t*-test). **j**–**l** Lipid peroxidation level (**j**), MDA level (**k**), and LIP level (**L**) in NC and PPM1F-KD A549 and PC9 cells with or without FRAX486 (0.5 μM, 24 h) under the treatment of RSL3 (1 μM, 24 h). (*n* = 3 biologically independent experiments, Student *t*-test). **m** WB showed downregulation of the expression of p-PAK in RARA-knockdown A549 and PC9 cells, while PAK showed no difference. Both PAK and p-PAK showed no difference in NC and TXN-OE cells. ns, not significant; *p < 0.05; **p < 0.01; ***p < 0.001;****p < 0.0001.

To validate that *RARA* inhibits ferroptosis in LUAD by promoting *TXN* and *PPM1F*, we generated A549 and PC9 cells overexpressing *TXN* and *PPM1F*, respectively (Fig. 6a). Cytotoxicity assays, MDA analysis, lipid peroxidation, and LIP assays demonstrated that respective overexpression of *TXN* or *PPM1F* made cells resistant to ferroptosis and partly reversed the ferroptosis-sensitizing effect rendered by *RARA* knockdown (Fig. 6b–e). The resistant effect was further enhanced when *TXN* and *PPM1F* were simultaneously overexpressed. Interestingly, our data revealed that overexpression of *TXN* confers greater resistance to ferroptosis than *PPM1F*, potentially due to the downstream action of *PPM1F* via kinases. We also assessed the sensitivity of cells co-overexpressing *TXN* and *PPM1F* to ferroptosis by MMP measurement and transmission electron microscopy. The results showed that mitochondria in *RARA*-KD A549 cells were significantly wrinkled and exhibited increased mitochondrial membrane density, lower Δψ, indicative of ferroptosis. However, these characteristic changes were not observed in cells co-overexpressing *TXN* and *PPM1F* (Fig. 6f, g). The ballooning phenotype was consistent with the above results (Fig. 6h).

Previous studies indicated that *PPM1F* inhibited ferroptosis by inhibiting PAK. We constructed *PPM1F*-KD A549 and PC9 cells (Supplementary Fig. 1f), and demonstrated that p-PAK was decreased in *PPM1F*-KD cells(Supplementary Fig. 1g). Also, *PPM1F*-KD was more sensitive to RSL3, and the enhanced effect could be reversed by DFO and fer-1(Fig. 6i). Lipid peroxidation, MDA, and LIP levels also increased in the *PPM1F*-KD group, which indicated that *PPM1F* serves as a ferroptosis suppressor (Fig. 6j–l). Furthermore, we used FRAX486 (0.5 μM, 24 h) to inhibit phosphorylation of PAK. As expected, dephosphorylation of PAK conferred resistance to RSL3 in NC and *PPM1F*-KD cells (Fig. 6i), with decreased lipid peroxidation (Fig. 6j), MDA (Fig. 6k) and LIP(Fig. 6l) levels, indicated that *PPM1F* inhibits ferroptosis via PAK dephosphorylation. The expression level of PAK showed no difference in NC and *RARA*-KD cells, but p-PAK was upregulated in *RARA*-KD A549 and PC9 cells, which demonstrated that *RARA* stimulated the expression of *PPM1F*, thereby promoting PAK dephosphorylation and inhibiting ferroptosis. Furthermore, the WB also showed that p-PAK was stable in *TXN*-OE cells compared to NC cells, which verified that there was no reciprocal regulation between TXN and PPM1F at the protein level (Fig. 6m). The above results demonstrate that *RARA* inhibits ferroptosis by promoting *TXN* and *PPM1F*, respectively.

### Pharmacological inhibition and knockdown of *RARA* enhance the effect of chemotherapy

**Fig. 7:**
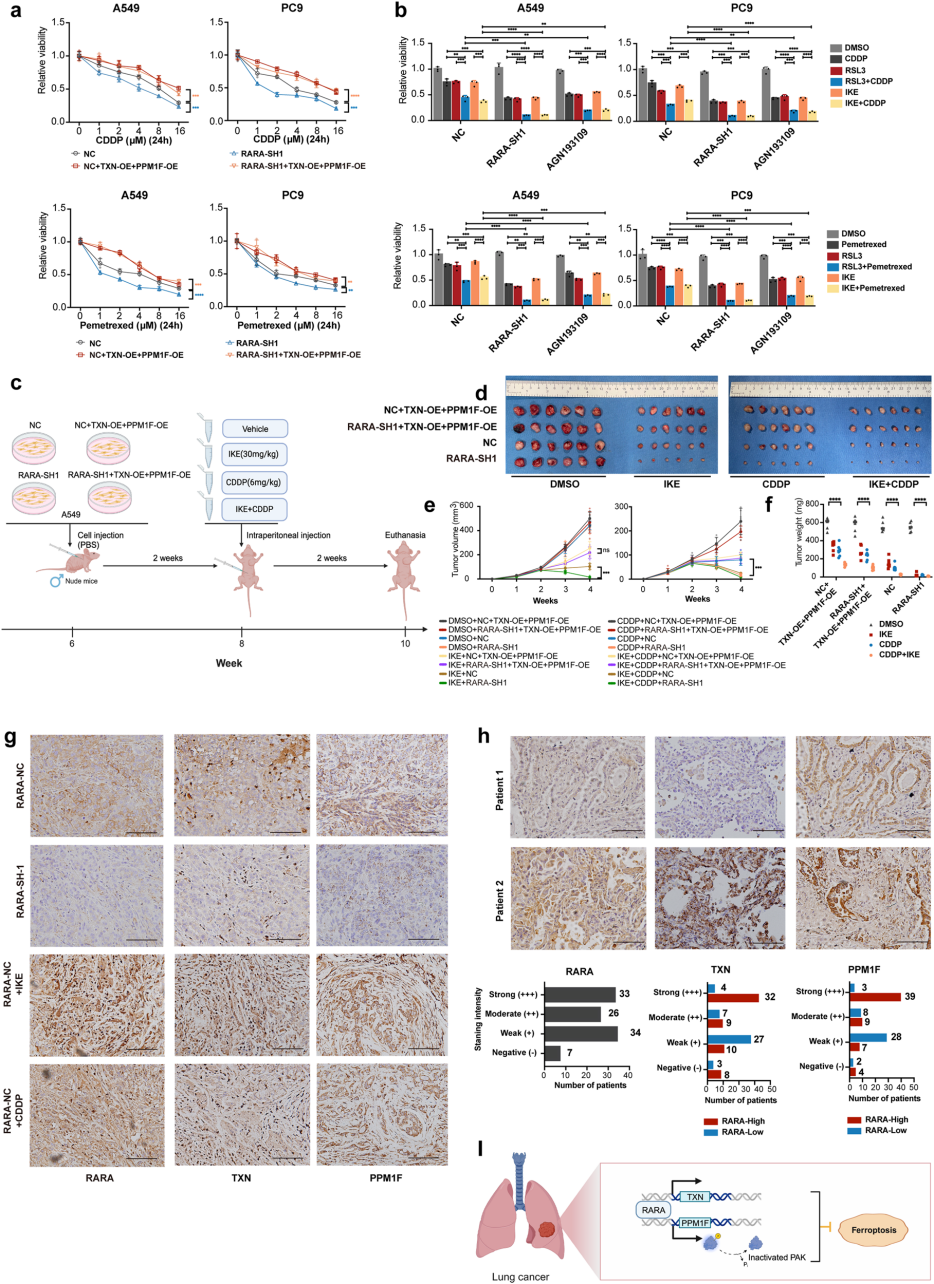
Pharmacological inhibition and genetic silence of RARA enhances the effect of chemotherapy. Knockdown of RARA confers sensitivity to IKE and could be rescued by TXN and PPM1F in vivo. **a** Dose-toxicity curves showed relative viability of NC and RARA-SH1 A549 and PC9 cells transfected with or without TXN-OE + PPM1F-OE upon a gradient dose CDDP or pemetrexed treatment for 24 h. (*n* = 3 biologically independent experiments, two-way ANOVA). **b** CCK8 assays showed the cell viability in NC, RARA-SH, inhibitor AGN193109 (5 μM, 24 h) groups of A549 and PC9 cells treated with RSL3 (2 μM) or IKE (5 μM), with or without CDDP (2 μM), pemetrexed (1 μM) for 24 h. (*n* = 3 biologically independent experiments, Student *t*-test). **c** Flow chart of the process of subcutaneous tumor formation in male nude mice and the treatment measures. **d** Representative tumors formed in nude mice by NC, RARA-SH1, NC + TXN-OE + PPM1F-OE, RARA-SH1 + TXN-OE + PPM1F-OE. Tumors were resected after 4 weeks. **e** Tumor volumes were measured weekly. (*n* = 6 biologically independent animals, two-way ANOVA). **f** The tumor weight of each group was weighed after 4 weeks. (*n* = 6 biologically independent animals, two-way ANOVA). **g** Representative images of immunohistochemistry staining of mice tumor. Scale bar:100 μm. **h** Representative images of immunohistochemistry staining of 100 LUAD patient samples. Scale bar:100 μm. **i** Mechanism diagram for RARA inhibiting ferroptosis through TXN and PPM1F. ns, not significant; *p < 0.05; **p < 0.01; ***p < 0.001; ****p < 0.0001.

Our previous study showed that ferroptosis also accounts for the cytotoxicity induced by chemotherapy pemetrexed and CCDP in LUAD^37^. So we explored the relationship between the effect of chemotherapy and ferroptosis mediated by *RARA*. Cytotoxicity assays showed that *RARA*-KD cells enhanced the effect of chemotherapeutic agents, including pemetrexed and CDDP in LUAD, while overexpression of *TXN* and *PPM1F* can did the opposite (Fig. 7a). Furthermore, FIN combined with chemotherapeutic agents showed more effect, and genetic or pharmacological inhibition of *RARA* enhanced the sensitivity (Fig. 7b). We conducted survival analysis in LUAD patients who received chemotherapy in TCGA and found that expression of *RARA*, as well as *TXN* and *PPM1F*, showed resistance to chemotherapy (Supplementary Fig. 1h). All the results above indicated that the *RARA* signaling pathway could be a potential target in combination therapy.

### *RARA* inhibits ferroptosis by promoting *TXN* and *PPM1F* in vivo

To further investigate the effects of *RARA* on ferroptosis in vivo, we carried out a xenograft tumor formation assay in male nude mice. Tumor xenografts were generated with NC, *RARA*-SH1, NC + *TXN*-OE + *PPM1F*-OE, and *RARA*-SH1 + *TXN*-OE + *PPM1F*-OE cell lines. Then mice were treated with either DMSO or IKE (30 mg/kg) or CDDP (6 mg/kg) or IKE (30 mg/kg) plus CDDP (6 mg/kg)via intraperitoneal injection every three days for six doses (Fig. 7c, created with BioRender.com). Treatment with IKE and CDDP significantly affected tumor growth compared to DMSO-treated controls. The combination treatment is more effective in slowing tumor growth compared to monotherapy. Furthermore, in both monotherapy and combination groups, *RARA* knockdown resulted in reduced tumor volume and weight, an effect that was reversed by overexpression of *TXN* and *PPM1F*, which was consistent with vitro experiments (Fig. 7d–f). The IHC staining showed that RARA, TXN, and PPM1F expression were all decreased in *RARA* knockdown mice tumors, and they showed the same tendency of upregulation upon IKE or CDDP treatment groups (Fig. 7g).

We further conducted IHC staining in 100 LUAD patients from our institution with high or low expression of RARA. The results showed that the expression of TXN and PPM1F showed the same tendency with RARA (Fig. 7h).

## Discussion

*RARA* is a member of the retinoic acid receptor family. Retinoic acid (RA) binds to RARA and plays a crucial role in cell growth and differentiation during embryonic development and adult physiology. Disruption of the RA signaling pathway is implicated in the development and progression of various malignancies^21^. ATRA is an active metabolite of vitamin A used in the treatment of various tumors, including lung cancer^24^, due to its effects on cell differentiation, proliferation, and apoptosis^38,39^. However, resistance to ATRA therapy can occur. Multiple studies have shown a close relationship between *RARA* and resistance to ATRA. Aberrant RARs phosphorylation and activity are associated with tumor resistance to ATRA^40^. In acute promyelocytic leukemia (APL), some rare *RARA* gene rearrangements exhibit resistance to ATRA and arsenic trioxide (ATO), including ZBTB16- RARA and STAT5B- RARA^41^. The signaling pathways of *RARA* and PI3K interact to activate the ERK pathway, leading to ATRA resistance^22^.

Ferroptosis is a distinct form of cell death characterized by iron-dependent ROS accumulation and phospholipid peroxidation. It is a nonapoptotic process that can be initiated by inhibiting the biosynthesis of glutathione or the activity of the glutathione-dependent antioxidant enzyme GPX4^42^. In recent years, ferroptosis induction has emerged as a promising therapeutic strategy for treating various tumors, including lung cancer^43^. In our research, we demonstrated that *RARA* inhibited ferroptosis in LUAD by promoting *TXN* and *PPM1F* transcription (Fig. 7i, created with BioRender.com).

We first discovered that the induction of ferroptosis is more difficult in A549 and PC9 cell lines following treatment with *RARA* activators (Ch55, AM580, and ATRA), while cells with *RARA* knockdown or treated with *RARA* inhibitor (AGN193109) are more sensitive to ferroptosis. Among the *RARA* activators tested, ATRA had the most pronounced effect, potentially due to its known antioxidant properties that inhibit lipid peroxidation in addition to its ability to activate *RARA*. These findings suggested that resistance to ATRA in treating lung cancer may be related to the regulation of ferroptosis by *RARA* and that inhibition of *RARA* could be a potential target for combination therapy.

TXN is a potent antioxidant that mediates many biological processes, including redox signaling and is closely related to tumor pathogenesis and therapy^44^. In tumor tissues, the upregulation of redox homeostasis in intrinsic tumor cells leads to increased levels of TXN and GSH, which reduce the production of ROS. This limits the effectiveness of anti-ferroptosis drugs^45^. Previous studies have demonstrated that overexpression of *TXN* can reverse the decrease in GPX4 and GSH levels and inhibit ferroptosis^29^. PAK is an effector kinase of small GTPases implicated in the development of various tumors due to its dysregulated activity or expression. PPM1F, expressed in various tumor cell lines, has been shown to dephosphorylate and downregulate PAK activity, with reports indicating its role in modulating breast cancer cell invasion^34^. Our study has confirmed that *TXN* and *PPM1F* are downstream targets of *RARA* and that there was no interaction between the two. *PPM1F* further inhibits ferroptosis by negatively regulating PAK. The inhibitory effect of *RARA* could be reversed by overexpression of *TXN* and *PPM1F* in both in vivo and in vitro experiments, further highlighting the vital role of inhibiting *RARA* in the treatment of inducing ferroptosis in cancer. Interestingly, the reversal effect of *PPM1F* overexpression on the increased sensitivity of *RARA*-KD cells to ferroptosis was more pronounced than that of *TXN* overexpression. This result may be attributed to *TXN* being an antioxidant and inhibiting ferroptosis. The relationship between *PPM1F* and ferroptosis has not been clearly studied, but its negative regulation of PAK, which has been shown to promote ferroptosis, suggests that the effect of *RARA* regulating *TXN* to inhibit ferroptosis is more pronounced.

However, several issues warrant further discussion and investigation. Firstly, although *RARA* has been proposed as a promising therapeutic target, no specific drug targeting *RARA* has been developed. Thus, developing effective and safe *RARA* inhibitors represents an important area for future research. *TXN* and *PPM1F* have been identified as critical regulators of ferroptosis in our study, but the mechanisms by which *TXN* modulates iron metabolism in LUAD remain unclear. Further investigation into the roles of *TXN* in LUAD is therefore warranted.

Tumors resistant to conventional therapies or prone to metastasis have been found to exhibit a high degree of vulnerability to ferroptosis^46,47^, highlighting the role of ferroptosis in treating patients with malignancies. Therefore, applying FIN in combination with chemotherapy or other antitumor drugs may provide an alternative solution for cancer patients. In our research, applying *RARA* inhibitor or knockdown of *RARA* increased the sensitivity of LUAD cells to ferroptosis and evaluated the effect of chemotherapy. Thus, targeting the *RARA* pathway may offer a novel approach to the comprehensive management of LUAD patients and provide more evidence for overcoming ATRA resistance.

## Methods

### Cell culture and compounds

We obtained four LUAD cell lines (A549, PC9, H23, H2122) and human embryonic kidney 293 (HEK293T) cells from the cell bank of the Chinese Academy of Sciences, which were cultured in DMEM high glucose medium (Hyclone, UT, USA) added with 10% fetal bovine serum (Every Green, Hangzhou, Zhejiang, China), 0.1 mg/ml streptomycin and 100 U/ml penicillin (Sangon Biotech, Shanghai, China). The cells were kept in a humidified environment with 95% air and 5% CO2. The RSL3, Imidazole Ketone Erastin (IKE), Erastin, Ferric ammonium citrate (FAC), *RARA* inhibitor AGN193109, *RARA* activator Ch55 and AM580, PAK inhibitor FRAX486, cisplatin (CDDP), and pemetrexed were acquired from Topscience (Shanghai, China) and were dissolved in dimethyl sulfoxide (DMSO). ATRA was purchased from Beyotime (Zhejiang, China). Other reagents, including ferrostatin-1(fer-1), Z-VAD-FMK(Z-VAD), deferoxamine (DFO), and necrosulfonamide (necro), were obtained from TargetMol (MA, USA).

### Transfection of siRNA, lentivirus, and gene overexpression

Two siRNAs (si*RARA*-1 and si*RARA*-2) targeting *RARA* and two negative control siRNAs (siNC-1and siNC-2), siRNA targeting *PPM1F* (si*PPM1F*) were designed by Ribobio (Ribobio, Guangzhou, China). They were transfected into cells using lipo8000 as transfection reagents (Beyotime, Zhejiang, China) and Opti-MEM (Thermo Fisher Scientific, MA, USA), following the manufacturer’s guidelines. The sequences of siRNAs are shown in Supplementary Table 2. Owing to their capacity to silence the *RARA* gene, two shRNAs were designed according to the si*RARA* sequences and subcloned into puromycin-resistant lentiviral vectors (Genechem, Shanghai, China). Subsequently, cells were transfected with the lentiviral vectors and subjected to puromycin selection for 48 h. Similarly, we constructed lentiviral vectors (Genechem) to mediate the overexpression of *TXN* and *PPM1F* and subsequently transfected them into cells.

### RNA extraction and quantitative real-time PCR (qRT-PCR)

We used TRIzol reagent (Tiangen, Beijing, China) for total RNA extraction according to the manufacturer’s instructions. The Hifair® II First-strand cDNA Synthesis Kit (YEASEN, Shanghai, China) was used to transcribe RNA into complementary DNA. We performed qRT-PCR using the Hieff® qPCR SYBR Green Master Mix (YEASEN) in the ABI QuantStudio 5 real-time PCR system (Thermo Fisher, Waltham, MA, USA). Samples were added in three replicates, and mRNA expression was assessed using the 2-ΔΔCT parameter and β-actin as endogenous controls. All primers were purchased at Sangon Biotech (Shanghai, China) and can be seen in Supplementary Table 2.

### Western blot (WB) experiments

Proteins were extracted from cells lysed on ice for 10 min using RIPA buffer (Beyotime, Shanghai, China) in conjunction with a protease and two phosphatase inhibitor cocktails (Topscience, Shanghai, China). Protein quantities were determined by BCA assay (YEASEN, Shanghai, China) and separated by sodium dodecyl sulfate-polyacrylamide gel electrophoresis (SDS-PAGE, YAMAY Biotech, Shanghai, China) before being transferred to polyvinylidene fluoride (PVDF) membranes (Millipore, Billerica, MA, USA). The membranes were then blocked with 5% non-fat milk for 1 h at room temperature and incubated overnight with a specific primary antibody at 4 °C. After three treatments with Tris-buffered saline-Tween (TBST) solution, the membranes were incubated with horseradish peroxidase (HRP)-labeled secondary antibody (1:2500, Beyotime) in the chamber for 1 h. Protein bands were observed using the Moon Chemiluminescence Kit (Beyotime), and the primary antibodies utilized in this study are listed in Supplementary Table 3.

### Cell viability assays

For cytotoxicity experiments, cells were seeded in 96-well plates at a density of 3000 cells per well and incubated for 24 h to allow for attachment and growth. After this initial incubation period, cells were pretreated with *RARA* activators and the inhibitor for 48 h before being exposed to various concentrations of RSL3 or IKE. Cell viability was assessed using the Cell Counting Kit-8 (Topscience), which measures the activity of cellular dehydrogenases as an indicator of cell metabolic activity. The optical density (OD) values were measured on a microplate reader to ascertain the relative number of viable cells in each well.

### Malondialdehyde (MDA) level measurement

The degree of ferroptosis was determined by analyzing the levels of MDA in each experimental group. Briefly, cells were cultured in 6-well plates, and cell lysates by RIPA buffer (Beyotime) were used to measure MDA levels using the MDA Assay Kit (Beyotime), following the manufacturer’s guidelines. The protein quantification was conducted using a BCA assay (YEASEN). The MDA content was evaluated colorimetrically by the reaction with thiobarbituric acid.

### Detection of Lipid Peroxidation (LPO) by BODIPY-C11

Cells cultured in 12-well plates were pretreated with various reagents and rinsed with phosphate-buffered saline (PBS, Beyotime). Cells are then resuspended in DMEM medium containing 200 μM BODIPY 581/591 C11 dye (Thermo Fisher, USA) for lipid peroxidation detection and incubated for 30 min at 37 °C. After rinsing three times with PBS, lipid peroxidation levels were assessed using the FITC channel of an Accuri 6 cytometer (BD, USA). Data were analyzed and displayed using FlowJo software (TreeStar, Woodburn, OR, USA).

### Detection of Mitochondrial Membrane Potential (MMP)

The assessment of mitochondrial membrane potential (MMP, Δψ) was conducted using the tetramethylrhodamine methyl ester (TMRE) dye (Thermo Fisher, USA). Cells were cultured in 12-well plates and underwent a 24 h exposure to each specific treatment. This was followed by a 30 min incubation period at 37 °C with 200 nM of the fluorescent dye TMRE. After centrifugation at 13,00 rpm for 5 min, the cells were resuspended in 400 μl PBS and were analyzed with a PE channel of an Accuri 6 cytometer (BD, USA). Data were analyzed using FlowJo software (TreeStar, Woodburn, OR, USA).

### Labile Iron Pool (LIP) assay

Labile iron pool (LIP) was measured using the calcein-acetoxymethyl ester (CA-AM) method. Cells were cultured in 12-well plates and collected with PBS. Then cells were incubated with 0.125 μM CA-AM (Thermo Fisher, USA) for 15 min at 37 °C. After washing with PBS, cells were treated with DFO (100 mM) or left untreated. Cells were analyzed with FACSAria III flow cytometry (BD). The mean fluorescence intensity (MFI) was calculated using FlowJo software (TreeStar, Woodburn, OR, USA). The difference in the MFI with and without DFO treatment reflects the LIP amount.

### Microscopy of “Ballooning” phenotype

Cells were cultured in 12-well plates. After different pre-treatments, light microscopy was used to observe the “ballooning” phenotype of morphological changes in cells^12^, as a specific feature of ferroptosis. Images were captured by Olympus IX71 microscope (Olympus).

### Chromatin immunoprecipitation (ChIP) analysis

Chromatin immunoprecipitation (ChIP) assays were performed using the SimpleChIP® Plus Enzymatic Chromatin IP Kit (Cell Signaling Technology, USA) according to the manufacturer’s protocol. Briefly, cells were treated with formaldehyde to crosslink DNA and associated proteins. Chromatin was then fragmented into 150–900 bp DNA/protein complexes using micrococcal nuclease digestion. Immunoprecipitation was performed using either control IgG or anti-*RARA* antibody (1:50 dilution, Cell Signaling Technology). Protein-DNA complexes were captured using protein G magnetic beads, and eluted chromatin was subjected to crosslink reversal. DNA fragments were purified using spin columns and quantified by qRT-PCR using the following primer pairs: TXN: Primer 1: F: CGTGGGCGTGTTCGATTCAG, R: AGGGCTTCGGCTCCTGTAAC; PPM1F: Primer 2: F: ACGGGATTAGAGGGCTGACG, R: ACCCATGTGAGCCTCGTCTC; Primer 3: F: AGAGAAGCAAGAGGCTGGTGACT, R: ACGGTGACTGCGGCCCTTTA.

### Dual-luciferase reporter analysis

Luciferase reporter gene assays were performed to investigate the regulation of *TXN* and *PPM1F* expression. The promoter region of *TXN* spanning from -2000 to +200 base pairs and *PPM1F*, spanning from -2000 – +300 base pairs relative to the transcription start site, as well as the corresponding mutant sequence, was cloned into the phy-811 luciferase reporter vector (Hanyin Technology, Shanghai, China). HEK-293T cells with either NC and *RARA* knockdown were co-transfected with *TXN*-WT, *PPM1F*-WT, or Mut plasmids and a Renilla luciferase reporter plasmid using Lipo8000 (Beyotime) at 60–80% confluency. Dual-luciferase reporter assays were performed using a Luciferase Reporter Gene Assay kit (Beyotime) according to the manufacturer’s instructions after 48 h post-transfection. Luciferase activity was measured using a FlexStation 3 Microplate Reader (Molecular Devices, San Jose, California, USA).

### Transmission electron microscopy

Treated cells cultured in 6 cm dishes were fixed with a solution containing 2.5% glutaraldehyde to preserve cellular structures by cross-linking proteins. After washing three times with 0.1 M phosphate buffer solution (PBS, pH 7.4) to remove excess glutaraldehyde, cells were further fixed with osmium acid in phosphate buffer. Following dehydration through a graded series of ethanol solutions and embedding in resin, samples were cured in a 60 °C oven for 48 h to allow the resin to harden. Ultrathin sections were then cut using an ultramicrotome and stained with lead citrate and uranyl acetate to enhance contrast. After drying overnight, sections were observed in a Hitachi transmission electron microscope (Hitachi, Japan) to visualize cellular ultrastructure.

### Animal experiments

The Animal Ethics Committee of Zhongshan Hospital, affiliated with Fudan University approved all animal experiments. We have complied with all relevant ethical regulations for animal use. Six-week-old male nude mice were purchased from Shanghai Jie Sijie Laboratory Animal Factory and housed in a pathogen-free environment within a laminar flow cabinet. A549 cells (2 × 10^6^) expressing NC, *RARA*-SH1, NC + *TXN-PPM1F*-OE, or *RARA*-SH1 + *TXN-PPM1F*-OE were resuspended in 100 μL PBS and subcutaneously injected into the right flank of each mouse. Once tumors reached approximately 50 mm^3^ in size, mice were randomly assigned to eight treatment groups. IKE (30 mg/kg) and CDDP(6 mg/kg) were administered intraperitoneally every three days for six times. Tumor size was measured weekly using vernier calipers, and tumor volume was calculated as (length ×  width^2^)/2. Mice were euthanized 4 weeks after tumor cell inoculation.

### Patients and tumor specimens

With informed consent, paraffin-embedded specimens of the tumor and adjacent healthy tissues were collected from 100 LUAD patients who underwent surgery from 2016 – 2019 at the Department of Thoracic Surgery, Zhongshan Hospital, Fudan University. All the patient specimens had comprehensive clinicopathology and follow-up information (Supplementary Table 1). The pathologic classification was based on World Health Organization (WHO) guidelines, and the tumor stage was assessed using the eighth edition of the International Union Against Cancer (UICC) Cancer Staging Manual according to the tumor/lymph node/metastasis (TNM) classification. The Zhongshan Hospital Research Ethics Committee at Fudan University approved this project (B2022-180R). All ethical regulations relevant to human research participants were followed.

### Immunohistochemistry (IHC)

According to the manufacturer’s protocol, paraffin-embedded tissues were dewaxed, hydrated, and stained using the GTVisionTM III Detection System (GeneTech, Shanghai, China). The antibodies can be seen in Supplementary Table 3. The detailed process can be found in the previous study^48^. PBS was employed as the negative control for the evaluation, which was determined based on staining intensity and the extent of positive staining. The staining intensity of colored cells was assessed using the immunoreactivity score (IRS) [IRS = Staining intensity × Proportion of positive-stained cells], with a scale of 0 (colorless/negative), 1 (light yellow/weak), 2 (dark yellow/moderate), and 3 (yellowish-brown/strong).

### Statistics and reproducibility

All data analysis was performed using GraphPad Prism 9 (GraphPad Software, La Jolla, CA) and R (version 4.1.2). Total RNA was extracted from cells using TRIzol reagent (TIANGEN) and subjected to sequencing. Reads were mapped to genes using TopHat (v.2.0.13) and HISAT2 (v.2.1.0), and raw data were normalized to fragments per kilobase of exon per million mapped reads (FPKM) for downstream analysis. Analysis and visualization of the differentially expressed genes were done using the limma and ggplot2 packages in R. Continuous variables were compared using Student’s *t*-test or two-way ANOVA, as appropriate. All tests were two-sided, and a *p*-value of less than 0.05 was considered statistically significant. The experiments were conducted in triplicate for every condition, and it was repeated at least 3 times.

### Supplementary information

Supplementary information

Description of Additional Supplementary Files

Supplementary data

## Data Availability

Source data supporting the findings of this study are available from the corresponding author upon reasonable request and Supplementary data. Numerical source data have been deposited in Figshare (10.6084/m9.figshare.25992559.v1). RNA-Seq data for A549 cells treated with FINs was deposited in Figshare (10.6084/m9.figshare.25835980). RNA-Seq data for A549 cells with RARA-KD was deposited in Figshare (10.6084/m9.figshare.25903963.v1). The plasmids information are available in Addgene, the ID number is 220375. The gating strategy for all flow cytometry (FACS) plots can be seen in Supplementary Fig. 2. The uncropped WB blots can be seen in Supplementary Fig. 3.
