# Supplementary material for: Retinoic acid receptor alpha inhibits ferroptosis by promoting thioredoxin and protein phosphatase 1F in lung adenocarcinoma
Source: Commun Biol. 2024 Jun 20;7:751. doi: 10.1038/s42003-024-06452-7 (PMC11190241; doi:10.1038/s42003-024-06452-7)
Supplement: Supplementary file 1 — Supplementary information [file 42003_2024_6452_MOESM1_ESM.pdf]

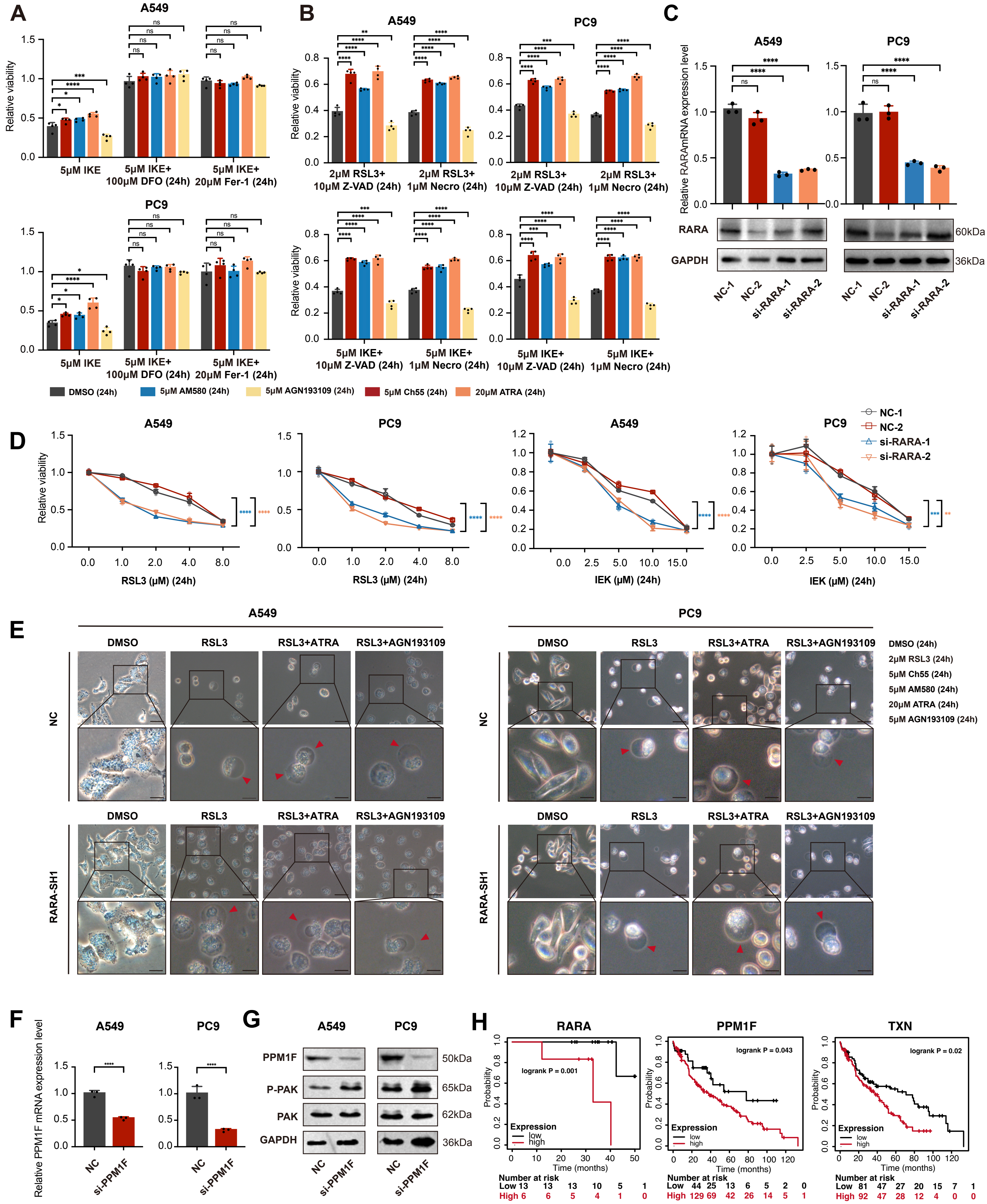
A
A549
Relative viability
5μM IKE
5μM IKE+ 100μM DFO (24h)
5μM IKE+ 20μM Fer-1 (24h)
PC9
B
A549
PC9
2μM RSL3+ 10μM Z-VAD (24h)
2μM RSL3+ 1μM Necro (24h)
5μM IKE+ 10μM Z-VAD (24h)
5μM IKE+ 1μM Necro (24h)
DMSO (24h)
5μM AM580 (24h)
5μM AGN193109 (24h)
5μM Ch55 (24h)
20μM ATRA (24h)
C
A549
PC9
Relative RARAmRNA expression level
RARA
GAPDH
60kDa
36kDa
NC-1
NC-2
si-RARA-1
si-RARA-2
D
A549
PC9
RSL3 (μM) (24h)
IEK (μM) (24h)
NC-1
NC-2
si-RARA-1
si-RARA-2
E
A549
PC9
DMSO
RSL3
RSL3+ATRA
RSL3+AGN193109
NC
RARA-SH1
DMSO (24h)
2μM RSL3 (24h)
5μM Ch55 (24h)
5μM AM580 (24h)
20μM ATRA (24h)
5μM AGN193109 (24h)
F
A549
PC9
Relative PPM1F mRNA expression level
NC
si-PPM1F
G
A549
PC9
PPM1F
P-PAK
PAK
GAPDH
50kDa
65kDa
62kDa
36kDa
NC
si-PPM1F
H
RARA
PPM1F
TXN
Probability
Time (months)
logrank P = 0.001
logrank P = 0.043
logrank P = 0.02
Expression
low
high
Number at risk
Low 13 13 13 10 5 1
High 6 6 5 4 1 0
Low 44 25 13 6 5 2 0
High 129 69 42 26 14 5 1
Low 81 47 27 20 15 7 1
High 92 47 28 12 4 0 0

Supplementary Figure 1 Pharmacological activation or inhibition and genetic knockdown of RARA changes sensitivity to ferroptosis in LUAD cells. a CCK8 assays to detect the cell viability of A549 and PC9 cells treated with only 24h-IKE (5 μM), IKE (5 μM) plus DFO (100 μM) or fer-1 (20 μM) after incubating with DMSO, Ch55 (5 μM), AM580 (5 μM) ATRA (20 μM) or AGN193109 (5 μM) for 24 h. (n=4 biologically independent experiments, Student t-test) **b** CCK8 assays to detect the cell viability of A549 and PC9 cells treated with only RSL3 (2 μM) or IKE (5 μM), RSL3 (2 μM) or IKE (5 μM) plus Z-VAD (10 μM) or necrosulfonamide (1 μM) for 24h after incubating with DMSO, Ch55 (5 μM), AM580 (5 μM) ATRA (20 μM) or AGN193109 (5 μM) for 24 h. (n=4 biologically independent experiments, Student t-test) **c** qRT-PCR and WB assays confirmed the knockdown effect of two siRNAs targeting RARA in A549 cells. (n=3 biologically independent experiments, Student t-test) **d** CCK8 assays to detect the cell viability of A549 cells transfected with NC (control siRNA) or si-RARA treated with a gradient dose of RSL3 or IKE for 24h. (n=4 biologically independent experiments, two-way ANOVA) **e** Light microscopy images showed the degrees of "ballooning phenotype" in NC or RARA-SH1-A549 and PC9 cells treated with DMSO and RSL3 (1 μM) for 24h after incubating with DMSO, Ch55 (5 μM), AM580 (5 μM) ATRA (20 μM) or AGN193109 (5μM) for 24 h. Scale bar:50μm. The zoomed-in figures, scale bar: 250μm. **f-g** qRT-PCR (**f**) and WB assays (**g**) confirmed the knockdown effect of siRNA targeting PPM1F in A549 cells. (n=3 biologically independent experiments, Student t-test) **g** WB assays showed that PPM1F dephosphorylated PAK. **h** Survival Analysis in LUAD patients received chemotherapy with different RARA, TXN and PPM1F expression. ns, not significant; *, $p < 0.05$; **, $p < 0.01$; ***, $p < 0.001$;****, $p < 0.0001$.

Supplementary Figure 2

Figure 2c

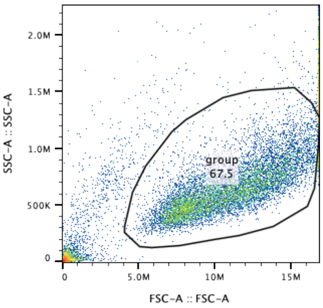

Figure 2d

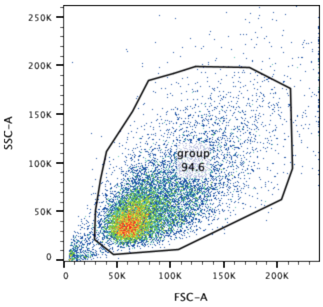

Figure 3e

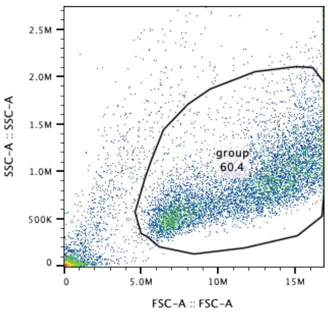

Figure 3f

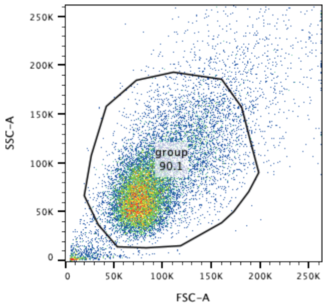

Figure 6d

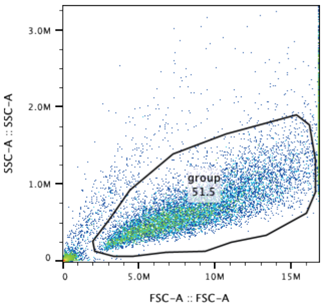

Figure 6f

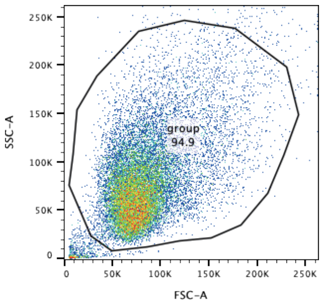

Figure 6j

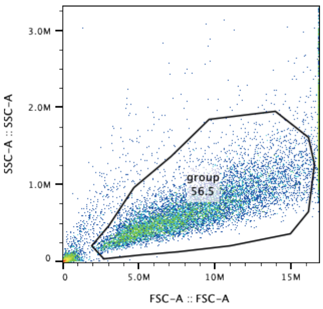

Supplementary Figure 3

Figure 1c

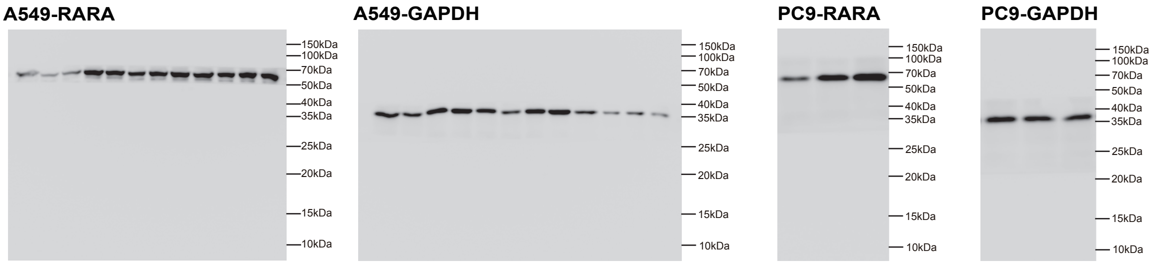

Figure 1d

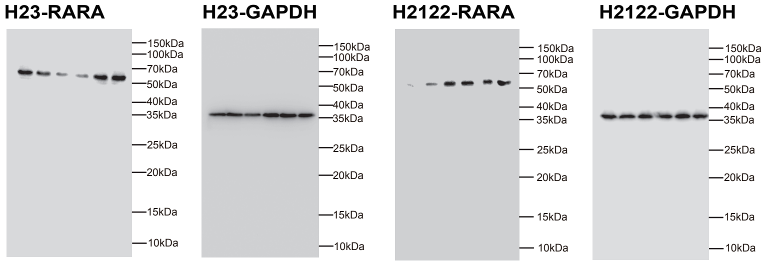

Figure 1e

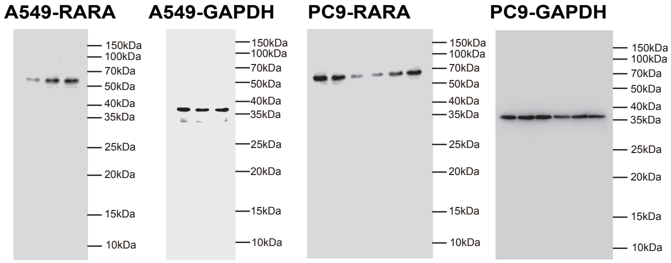

Figure 3a

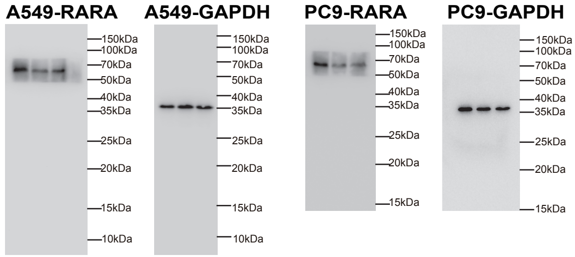

Figure 4e

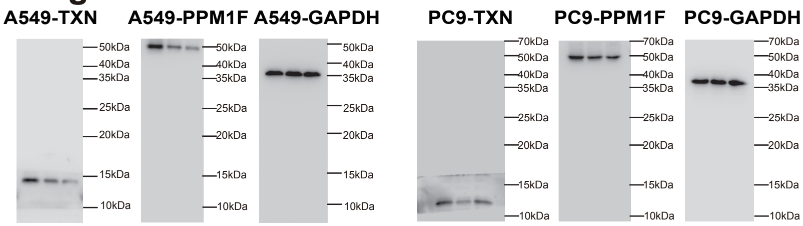

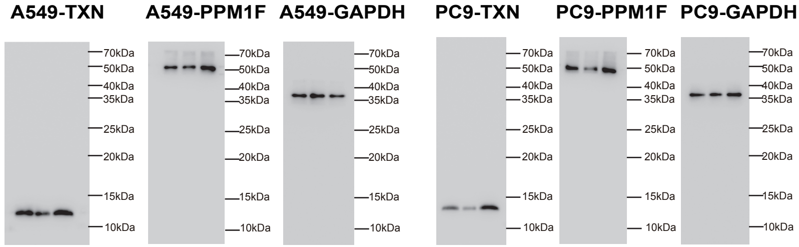

Figure 4g

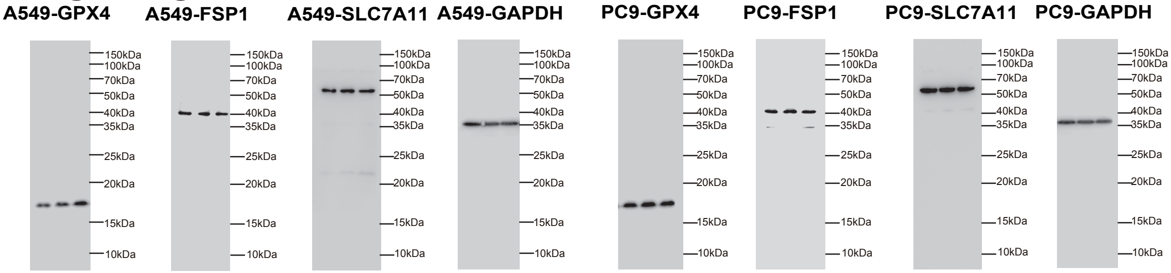

Supplementary Figure 4

Figure 6a

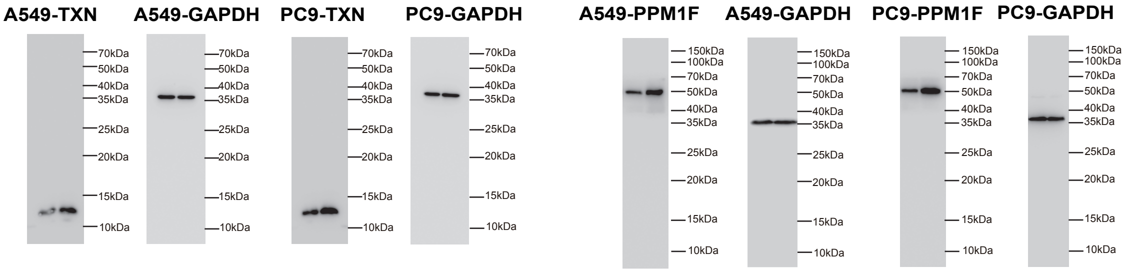

Figure 6m

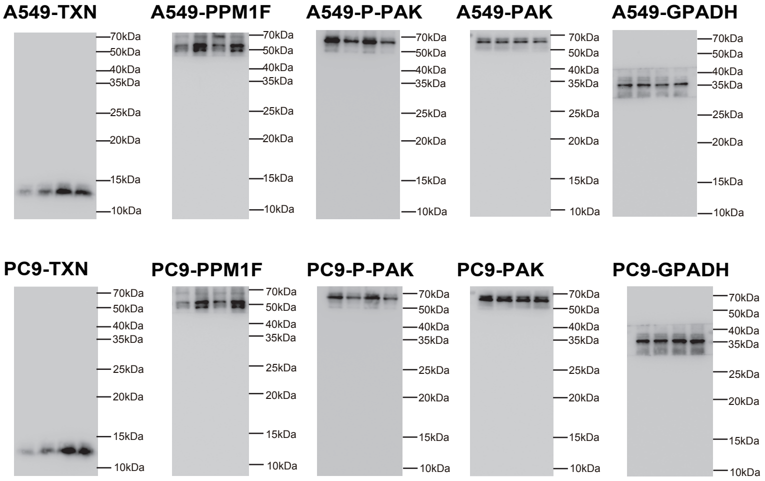

Supplementary Figure 1c

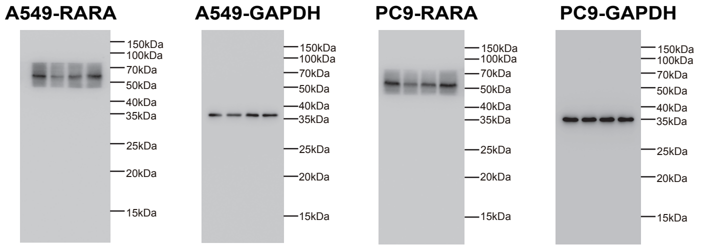

Supplementary Figure 1g

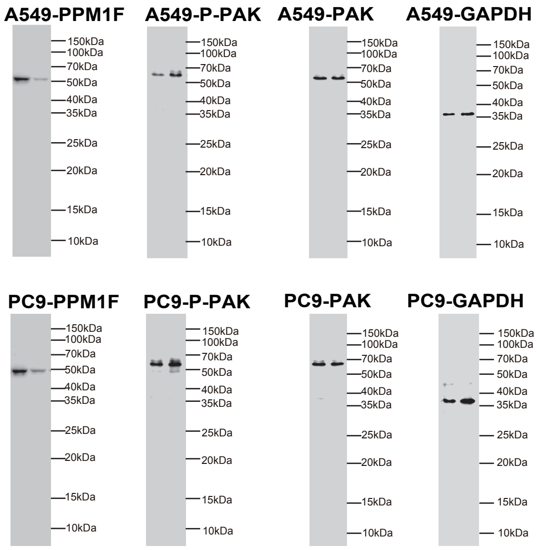

Supplementary Table 1 Summary of clinical information by groups of RARα expression

	High<br>*N=59*	Low<br>*N=41*	p-value
**Age**			0.967
<60	23 (39.0%)	17 (41.5%)	
>60	36 (61.0%)	24 (58.5%)	
**Gender**			0.898
Female	34 (57.6%)	25 (61.0%)	
Male	25 (42.4%)	16 (39.0%)	
**Stage**			0.075
I+II	48 (81.4%)	26 (63.4%)	
III	11 (18.6%)	15 (36.6%)	
**T**			1.000
T1+T2	45 (76.3%)	32 (78.0%)	
T3+T4	14 (23.7%)	9 (22.0%)	
**N**			0.327
N0	26 (44.1%)	23 (56.1%)	
N1-N2	33 (55.9%)	18 (43.9%)	
**TXN expression**			<0.001
High	41 (69.5%)	11 (26.8%)	
Low	18 (30.5%)	30 (73.2%)	
**PPM1F expression**			0.002
High	34 (57.6%)	10 (24.4%)	
Low	25 (42.4%)	31 (75.6%)	

Supplementary Table 2 The sequences of all the siRNAs and primers used in this study

siRNA	Sequence
siRARα-1	5’-GTGAGAAACGACCGAAACA-3’
siRARα-2	5’-ATTACTGACCTGCGAAGCA-3’
siPPM1F	5’-GTTTCACAGCTGCTACAGACA-3’
**Primer**	**Sequence**
GAPDH-F	5’-AGAAGGCTGGGGCTCATTTG-3’
GAPDH-R	5’-AGGGGCCATCCACAGTCTTC-3
ACTB-F	5’-CTGGGACGACATGGAGAAAA-3’
ACTB-R	5’-AAGGAAGGCTGGAAGAGTGC-3’
RARα-F	5’-AGAGCAGCAGTTCTGAAGAGATAGTG-3’
RARα-R	5’-CATAGTGGTAGCCTGAGGACTTGTC-3’
TXN-F	5’-GTGAAGCAGATCGAGAGCAAG-3’
TXN-R	5’-CGTGGCTGAGAAGTCAACTACTA-3’
PPM1F-F	5’-GGGATTCCCAGGTCATTTTGG-3’
PPM1F-R	5’-TCCTGCCGTTCTGGTCTGT-3’
HMOX1-F	5’-AAGACTGCGTTCCTGCTCAAC-3’
HMOX1-R	5’-AAGACTGCGTTCCTGCTCAAC-3’
GCLC-F	5’-GGAGGAAACCAAGCGCCAT-3’
GCLC-R	5’-CTTGACGGCGTGGTAGATGT-3’

Supplementary Table 3 The primary antibodies used in this study

Primary antibodies	Producer	ID/ Number	Dilution in WB	Dilution in IHC
GAPDH	Beyotime	AG019-1	1:2000	-
RARα	Abclonal	A19551	1:1000	1:50
TXN	Abclonal	A4024	1:1000	
TXN	Abways	CY6680		1:50
PPM1F	Abcam	ab200394	1:500	1:50
GPX4	Abways	CY6959	1:1000	
FSP1	Abclonal	A22278	1:1000	
SLC7A11	Abways	CY7046	1:1000	
PAK	Abways	AY0665	1:1000	-
p-PAK	Abways	CY5349	1:1000	-
