# Supplementary material for: Retinoic acid receptor alpha inhibits ferroptosis by promoting thioredoxin and protein phosphatase 1F in lung adenocarcinoma
Source: Commun Biol. 2024 Jun 20;7:751. doi: 10.1038/s42003-024-06452-7 (PMC11190241; doi:10.1038/s42003-024-06452-7)
Supplement: Supplementary file 2 — Description of Additional Supplementary Files [file 42003_2024_6452_MOESM2_ESM.pdf]

Description of Additional Supplementary Files

File name- Supplementary Data

File description- source data behind the graphs in the paper.
